# Selection of the optimal intensity normalization region for FDG-PET studies of normal aging and Alzheimer’s disease

**DOI:** 10.1038/s41598-020-65957-3

**Published:** 2020-06-09

**Authors:** Scott Nugent, Etienne Croteau, Olivier Potvin, Christian-Alexandre Castellano, Louis Dieumegarde, Stephen C. Cunnane, Simon Duchesne

**Affiliations:** 1 CERVO Research Centre, Quebec Mental Health Institute, Quebec, Canada; 2 Research Center on Aging, Health and Social Sciences Center, Geriatrics Institute, Sherbrooke, Canada; 3 Radiology and Nuclear Medicine Department, Université Laval, Québec, Canada

**Keywords:** Neural ageing, Ageing

## Abstract

The primary method for measuring brain metabolism in humans is positron emission tomography (PET) imaging using the tracer ^18^F-fluorodeoxyglucose (FDG). Standardized uptake value ratios (SUVR) are commonly calculated from FDG-PET images to examine intra- and inter-subject effects. Various reference regions are used in the literature of FDG-PET studies of normal aging, making comparison between studies difficult. Our primary objective was to determine the optimal SUVR reference region in the context of healthy aging, using partial volume effect (PVE) and non-PVE corrected data. We calculated quantitative cerebral metabolic rates of glucose (CMRg) from PVE-corrected and non-corrected images from young and older adults. We also investigated regional atrophy using magnetic resonance (MR) images. *FreeSurfer* 6.0 atlases were used to explore possible reference regions of interest (ROI). Multiple regression was used to predict CMRg data, in each *FreeSurfer* ROI, with age and sex as predictors. Age had the least effect in predicting CMRg for PVE corrected data in the pons (*r*^2^ = 2.83 × 10^−3^, *p *= 0.67). For non-PVE corrected data age also had the least effect in predicting CMRg in the pons (*r*^2^ = 3.12 × 10^−3^, *p *= 0.67). We compared the effects of using the whole brain or the pons as a reference region in PVE corrected data in two regions susceptible to hypometabolism in Alzheimer’s disease, the posterior cingulate and precuneus. Using the whole brain as a reference region resulted in non-significant group differences in the posterior cingulate while there were significant differences between all three groups in the precuneus (all *p *< 0.004). When using the pons as a reference region there was significant differences between all groups for both the posterior cingulate and the precuneus (all *p* < 0.001). Therefore, the use of the pons as a reference region is more sensitive to hypometabism changes associated with Alzheimer’s disease than the whole brain.

## Introduction

### Semi-quantitative analysis of cerebral glucose uptake

Positron emission tomography (PET) imaging using the tracer ^18^F-fluorodeoxyglucose (FDG) is the primary method used to measure *in vivo* brain metabolism of the primary energy substrate used by the brain, namely, glucose. FDG-PET images are commonly expressed in units of standardized uptake values (SUV), a semi-quantitative method that allows the examination of inter-subject effects. SUV is calculated as the ratio of tissue activity concentration and administration dose, divided by body weight; its major advantage being that it does not require blood sampling or dynamic imaging as quantitative imaging does. The SUV ratio (SUVR) is another semi-quantitative method that normalizes for differences in body weight, administration dose and tracer plasma clearance by dividing tracer uptake by that of an entire normalization region. The optimal SUVR reference region is to be chosen based on its isometabolic activity between control and condition groups, on the assumption that it is either least affected by the disease/paradigm or remains independent of its processes. Image activity is then expressed as a ratio relative to uptake in this reference region. The choice of reference region may change depending on the population being studied, the specific processing pipeline used, and the availability of possible reference regions masks. Variations can affect the conclusions of a study, as different regions will likely change individual statistical maps.

## Hypometabolism in cognitively normal aging

At present, there is a lack of consensus regarding the choice of reference region for FDG studies of normal aging. Choosing a reference region in this context is difficult due to the presence of widespread hypometabolism^1^. In addition to the requirement of limited metabolic and volumetric variability during aging, the optimal reference region should not be susceptible to external physiological stimuli, such as visual, tactile or auditory, and should be reliably segmented. Zhang, *et al*.^2^ examined possible reference regions during normal aging. The authors compare all automated anatomical labeling (AAL) regions^3^ and calculated an index of scatter for each region. Regions with the highest consistency were highlighted as the most appropriate for normalization in normal aging. The authors found that normalization with the paracentral lobule had the least scatter across age, followed by the cerebellar tonsil^2^. Both regions performed significantly better than the global mean. Of note, the authors did not include brainstem regions in their analysis, nor did they correct for any possible effects of atrophy, since no magnetic resonance (MR) images were available^2^. In addition, the authors noted in their limitations that they only used intensity normalized data and that quantitative techniques would be necessary to calculate the true trajectory of brain glucose uptake during aging. Other authors have reported using various reference regions across studies of normal aging, which are summarized in Table 1. From this survey, one can appreciate the lack of consensus regarding the most appropriate reference regions for studies on normal aging.

**Table 1:** Variability in choices of reference regions across healthy aging studies.

Manuscript	Reference region chosen	PVE correction
Ibanez, *et al*.^20^	Whole brain	Yes
Yanase, *et al*.^21^	Whole brain	Yes
Iseki, *et al*.^22^	Whole brain	No
Kalpouzos, *et al*.^23^	Whole brain	Yes
Curiati, *et al*.^24^	Whole brain	Yes
Knopman, *et al*.^25^	Pons	Yes
Ewers and Batteas^26^	Pons	Yes
Berti, *et al*.^17^	Pons	No
Greve, *et al*.^16^	Pons	Yes
Krell-Roesch, *et al*.^27^	Pons and cerebellum vermis	No
Gardener, *et al*.^28^	Entire cerebellum	No
Bonte, *et al*.^29^	Entire cerebellum	Yes
Shokouhi, *et al*.^30^	Cerebellar grey	No
Jiang, *et al*.^19^	Paracentral lobule	No

Partial volume effect (PVE).

In general, the aforementioned studies that attempted to address the question of the optimal reference region suffered from a number of limitations. Principally, studies either did not take into account the effects of atrophy, mainly because they did not include MR images in their studies; or did not include the brainstem in their analysis, possibly due to limited signal to noise ratio (SNR) or due to an inability to automatically segment this region of the brain.

## Objective and contributions

The primary objective of this study was to overcome these limitations in order to determine the most appropriate reference regions for semi-quantitative reporting of FDG-PET in studies of cognitively normal aging. We compared young and older adults using MR structural data and quantitative metabolic FDG-PET images with, and without partial volume effect (PVE) correction to help eliminate the effects of atrophy on estimates of brain glucose uptake^4^.

## Methods

### Participants

Ethical approval for this study was obtained from the ethics committees of the Centre de santé et de services sociaux – Institut universitaire de gériatrie de Sherbrooke and the Centre hospitalier universitaire de Sherbrooke. The study was conducted in accordance with the Declaration of Helsinki. All participants provided written informed consent prior to study entry. Participants were between either 18–30 years old (Younger group; n = 30), or 65–85 years old (Older group; n = 29; *cf*. Table 2). All participants underwent a pre-screening visit, which included analysis of blood collected after an overnight fast, and completion of a medical history questionnaire including the use of prescription medications. Exclusion criteria included diabetes or glucose intolerance (fasting plasma glucose ≥6.1 mM or glycated hemoglobin [HbA1c; ≥6.5%], according to World Health Organization recommendations^5^, evidence of overt heart, liver or renal disease, indication of cognitive impairment (Mini-mental state examination score <26/30), smoking, or untreated dyslipidemia, hypertension, or thyroid disease. In order to validate our findings, we used Control and Alzheimer disease patients from the Alzheimer’s Disease Neuroimaging Initiative (ADNI) database (adni.loni.usc.edu). The ADNI was launched in 2003 as a public-private partnership, led by Principal Investigator Michael W. Weiner, MD. The primary goal of ADNI has been to test whether serial magnetic resonance imaging (MRI), positron emission tomography (PET), other biological markers, and clinical and neuropsychological assessment can be combined to measure the progression of mild cognitive impairment (MCI) and early Alzheimer’s disease (AD).

**Table 2:** Characteristics of study participants.

	Young		Older		*p* value
	Mean	SD	Mean	SD	
Number/group	29		30		
Age (y)	26	4	72	5	≤0.001
Sex (M/F)	13/17	—	10/19	—	
Education (y)	16	2	14	4	0.003
Height (cm)	171	10	163	14	0.017
Weight (kg)	69	13	74	16	
Body mass index (kg/m^2^)	23	3	28	5	≤0.001
Total body fat (%)	27	9	36	9	≤0.001
**Fasting plasma measurements**					
Glucose (mM)	4.8	0.4	5.2	0.5	0.003
Cholesterol (mM)	4.1	0.8	5.1	1.3	0.002
HDL cholesterol (mmol/L)	1.5	0.3	1.5	0.3	
LDL cholesterol (mmol/L)	2.2	0.7	3.1	1.1	0.002
Triglycerides (mM)	0.9	0.5	1.2	0.4	0.005
Free fatty acids (mM)	0.8	0.3	0.9	0.4	
Insulin (IU/L)	4.2	1.8	5.2	4.5	
Hemoglobin A1_c_ (%)	5.2	0.2	5.7	0.2	≤0.001
Albumin (g/L)	44	3	43	2	
Thyroid stimulating hormone (mIU/L)	2.2	0.8	2.5	0.9	
Creatinine (µmol/L)	73	15	74	17	

*P*-values were calculated using unpaired *t*-tests and a chi-square test was used to evaluate sex differences between the two groups, uncorrected for multiple comparisons, *p ≤ 0.05.

### Plasma metabolites and body fat content

All plasma metabolites were measured using an automated clinical chemistry analyzer (Dimension Xpand Plus; Siemens Healthcare Diagnostics, Deerfield, IL, USA). Plasma insulin was analyzed by commercial enzyme-linked immunosorbent assay (Alpco, Salem, NH, USA) with a Victor X4 multilabel plate reader (Perkin Elmer, Woodbridge, ON, Canada). TSH was measured using electrochemiluminescence (ECL) sandwich immunoassay. Percent body fat was calculated with dual X-ray absorptiometry (DXA; GE Lunar Prodigy).

### PET acquisition

Brain PET scans were performed on a Philips Gemini TF PET/CT scanner (Philips Medical System, Eindhoven, The Netherlands) using a dynamic list mode acquisition, as described previously^6^. Time-of-flight was enabled, with an isotropic voxel size of 2 mm^3^, field-of-view of 25 cm, and an axial field of 18 cm. Time frames were allocated according to 12 × 10 s, 8 × 30 s, 6 × 4 min, and 6 × 5 min, for a total scan length of 60 minutes. After breakfast, each participant fasted for 6–7 hours before scanning, which was performed at around 3 o’clock pm. Each participant’s head was positioned in the headrest and gently restrained with straps in a dark quiet environment. An indwelling venous catheter was introduced into a forearm vein with the arm placed in a hand warmer at 44 °C^7^. A second catheter was placed in the contralateral forearm vein to infuse 5 mCi of FDG over 20 seconds using an infusion pump. Blood samples were obtained at 3, 8, 16, 24, 35 and 55 minutes after FDG infusion. Radioactivity in plasma samples was counted in a gamma counter (Cobra, Packard, United States) and cross-calibrated with the PET scanner, as described previously^1^.

### MR images

For all participants, T_1_-weighted anatomical MR images were acquired on a 1.5 Tesla scanner (Sonata, Siemens Medical Solutions, Erlangen, Germany) with a gradient echo sequence (repetition time/echo time: 16.00/4.68 ms; 20° flip angle; 1 mm^3^ isotropic voxel size; 256 × 240 × 192 mm field of view; matrix size 256 × 256 × 164).

### Cerebral metabolic rate of glucose estimation

PET images were first motion-corrected and then co-registered to the individual’s anatomic T1-weighted MR image using a rigid transformation with the cross-modality 3D image fusion tool implemented in PMOD 3.8 (PMOD Technologies Ltd., Zurich, Switzerland). Co-registered PET images were then corrected for partial volume effects using the modified Müller-Gartner method^8^, implemented in PMOD 3.8. Arterial input functions were determined by tracing regions of interest (ROIs) on the internal carotid arteries with the aid of co-registered MR images as previously validated in humans^9^. The input functions were partial volume-corrected and calibrated against the corrected radioactivity in blood samples obtained during each PET scan, as described previously^10^. The first part of the time-activity curve was modeled by a linear function to reflect the increasing blood [18F]-FDG concentration during the automated injection of the tracer. The initial linear function, blood-sampled and image-derived input functions were concatenated and interpolated through a tri-exponential decay fit (PMOD 3.7 Technologies Ltd., Zurich, Switzerland). The lumped constant used to calculate the quantitative cerebral metabolic rate of glucose (CMRg) was set to 0.80^11^. CMRg was expressed as µmol/100 g/min using the graphical Patlak model^12^. CMRg was calculated as the product of the rate constant (*K*) for the tracer uptake and the plasma concentration of glucose divided by the lumped constant. Finally, quantitative CMRg data were averaged within each anatomical ROI PVE corrected and non-PVE corrected images, as defined below.

### Cerebellar, cerebral, and brainstem volumes

Possible normalization regions were either defined using the *FreeSurfer* image analysis suite (http://surfer.nmr.mgh.harvard.edu/). Cortical reconstruction and volumetric segmentation were performed using version 6.0. In summary, *FreeSurfer* takes the T1-weighted MRI and follows a number of pre-processing steps (motion correction and average of multiple volumetric T1-weighted images; removal of non-brain tissue; automated affine and non-linear transformation to pseudo-Talairach space; intensity normalization) before segmenting subcortical white and deep grey matter volumetric structures, tessellate the grey matter and white matter boundary, correct topology, and deform the surfaces to optimally place the grey/white and grey/cerebrospinal fluid boundaries^13^. Following this process, cortical structures are identified and then applied to FDG-PET images in order to identify 84 possible normalization regions and compute averages within each.

### Data presentation and statistical analysis

Laterality was examined for each brain region. In cases where no significant differences existed the left and right brain regions were grouped together. Multiple regression was used to predict CMRg and volumetric data using age and sex as predictors using the R package relaimpo.

## Results

### Participant characteristics

Demographics for each group are shown in Table 2. As expected, there was a significant difference in age between the Younger and Older group (p ≤ 0.001). The Younger group had significantly more years of education than the Older group (p = 0.003); they were also taller (p ≤ 0.017), had a lower BMI (p ≤ 0.001) and less total body fat (p ≤ 0.001). In addition, plasma parameters showed that the Younger group had lower plasma glucose (p = 0.003), cholesterol (p = 0.002), LDL (p = 0.002), triglycerides (p = 0.005), and hemoglobin A1c (p ≤ 0.001). All the blood results were in the normal range according to the laboratory reference values from the Centre hospitalier universitaire de Sherbrooke (Sherbrooke, QC).

### Non-PVE corrected brain glucose uptake

**Figure 1:**
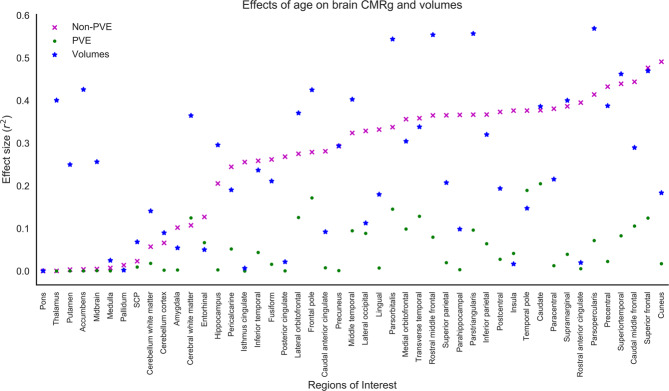
Plot of non-partial volume effect (PVE) and PVE corrected cerebral metabolic rate of glucose (CMRg) and volume effect size (*r*^*2*^). Plot is arranged in increasing order of *r*^*2*^ for each FreeSurfer region of interest.

Multiple regression was used to predict CMRg for non-PVE corrected images with age and sex as predictors. Figure 1 shows the effect sizes for all regions listed in order of least to most significant effects for non-PVE corrected images. Age had the least effect in predicting non-PVE corrected CMRg in the pons (*r*^2^ = 3.12 × 10^−3^, *p* = 0.67).

### PVE corrected brain glucose uptake

Multiple regression was used to predict CMRg data, in each *FreeSurfer* ROI, with age and sex as predictors. Figure 1 shows the effect sizes for all regions. Age had the least effect in predicting PVE corrected CMRg in the pons (*r*^2^ = 2.83 × 10^−3^, *p* = 0.67).

### FreeSurfer brain volumes

Multiple regression was used to predict brain volumes, in each *FreeSurfer* ROI, with age and sex as predictors. Figure 1 shows the effect sizes for all regions. Age had the least effect in predicting brain volumes in the pons (*r*^2^ = 3.07 × 10^−2^, *p* = 0.60).

### AD susceptible regions

**Figure 2:**
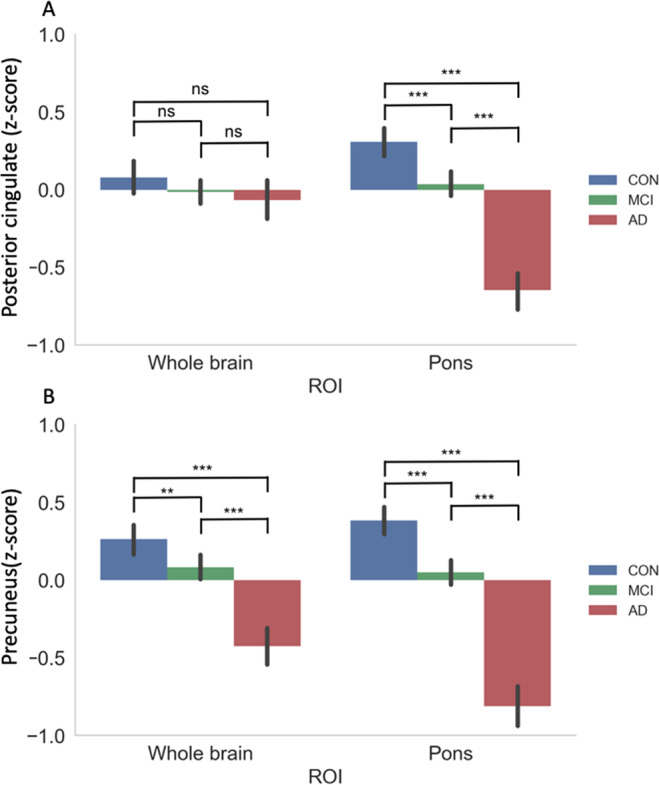
Effects of whole brain and pons reference regions on group differences between healthy controls, mild cognitive impairment (MCI), and Alzheimer’s disease (AD) patients using PVE corrected data. Two regions susceptible to hypometabolism in Alzheimer’s disease, the posterior cingulate (**A**) and precuneus (**B**), were analyzed. Cerebral metabolic rate of glucose (CMRg) are displayed as z-scores. ** *p* < 0.01, *** *p* < 0.001.

We examined differences in the effects of whole brain and pons reference regions on group differences between healthy controls, mild cognitive impairment (MCI), and Alzheimer’s disease (AD) patients using PVE corrected data (Fig. 2). Two regions susceptible to hypometabolism in Alzheimer’s disease, the posterior cingulate and precuneus, were analyzed. Using the whole brain as a reference region resulted in non-significant group differences in the posterior cingulate while there were significant differences between all three groups in the precuneus (all *p* < 0.004). When using the pons as a reference region there was significant differences between all groups for both the posterior cingulate and the precuneus (all *p* < 0.001).

## Discussion

### Optimal reference region

Authors have employed a variety of reference regions in their studies of normal aging. Commonly, these have included the cerebellum, pons, brainstem, sensorimotor cortex, or the whole brain. For consistency, a verified reference region should be employed to optimize comparability between studies. In this study, we aimed to determine the region that was the most consistent during aging. We therefore used multiple regression to predict CMRg and volumetric data with age and sex as predictors to determine in which regions age least significantly predicted CMRg.

For non-PVE corrected data the pons had the smallest age effects of all brain regions (Fig. 1). Therefore, utilization of the pons in calculation of SUVR in non-PVE corrected data will introduce the least amount of variability and lead to increased sensitivity. In addition, the pons was the regions that was least affected by brain atrophy during aging (Fig. 1). In order to interpret the non-PVE corrected FDG-PET data it is necessary to consider the effects of atrophy on estimates of brain glucose uptake. Regions with large rates of atrophy are likely to introduce variability into estimates of brain metabolism due to the introduction of varying degrees of partial volume effect and should be avoided.

We also examined FDG-PET images that were PVE corrected in order to help eliminate the effects of atrophy. We determined that the pons remained the region which was least affected by aging (Fig. 1).

### Comparison of results

With respect to PVE and non-PVE corrected studies, our results suggest that the pons is the ideal reference region. Several studies have previously used the pons as a reference region in studies of normal aging, however they either give no indication why^14,15^, use it because previous papers have^16^, or cite Minoshima *et al*.^17^. However, Minoshima, *et al*.^18^ did not study normal aging, the authors only studied a group of older adults with AD and compared glucose uptake to a control group of older adults (mean age 68 years). Therefore, they did not have the ability to examine changes in brain glucose uptake during normal aging. To our knowledge, no studies of strictly normal aging have shown that the pons should be used as a reference region. One limitation of the pons is that it is amongst the most inferior regions scanned and possibly within the outer extremes of the axial PET scanner field of view. Optimal participant placement is crucial to help in alleviating any loss of scanner sensitivity in this region.

The cerebellum is also commonly used for image normalization. Our results found significant effects of age in the cerebellum cortex (*r*^2^ = 0.06, *p* = 0.05) for non-PVE corrected data. PVE corrected cerebellar activity was found to not be significantly associated with age. The cerebellar cortex is also significantly affected by aging (*r*^2^ = 0.09, *p* = 0.02), while cerebellar white matter is not. Therefore, studies that want to use the cerebellum to normalize FDG-PET should use only the cerebellum white matter and not include cerebellum grey matter.

Many studies use the whole brain for normalization, however we found significant effects of age on whole brain glucose uptake for PVE corrected (*r*^2^ = 0.07, *p* = 0.04), non-corrected (*r*^2^ = 0.25, *p* < 0.001), as well as brain volume (*r*^2^ = 0.47, *p* = 0.007). Therefore, we do not recommend the use of whole brain normalization using PVE or non-PVE corrected data.

Previous studies have compared various reference regions to the whole brain. Zhang, *et al*.^2^ found that the paracentral lobule and cerebellar tonsil had significantly greater coherency when compared with global mean normalization. In addition, normalization to the global mean may result in regions of apparent hypermetabolism in regions that are relatively spared of metabolic decline during aging. Such regions often include the primary sensorimotor cortices, cerebellum, basal ganglia, and the brainstem, all of which have been proposed as possible normalization regions. Generally, normalization using these areas rather than whole brain activity will result in an increase in test sensitivity due to their improved variability.

Previous studies on normal aging have used the paracentral cortex as a reference region^19^ and this region has been proposed in a recent study by^2^. We found that age was significantly associated with CMRg in the paracentral cortex for non-PVE corrected (*r*^2^ = 0.38, *p* < 0.001) and brain volume data (*r*^2^ = 0.22, *p* = 0.01). Therefore, we would strongly caution against using the paracentral cortex as a reference region for non-PVE corrected data. The paracentral cortex was not significantly associated with age using PVE corrected data, therefore, it may be possible to use it in this regards.

### Strengths

This study includes quantitative CMRg FDG-PET data. Previous studies have not used quantitative measure to evaluate the optimal reference region. Additionally, this study includes magnetic resonance measures. This has the benefit of helping to eliminate the effects of atrophy through the correction of partial volume effects. Previous studies evaluating reference regions in normal aging did not correct for partial volumes or take into account the effects of atrophy.

### Limitations

The sample size used in our study raises the possibility of differences between our sample and the population arising simply by chance. Additionally, since the focus was on cognitively healthy aging, our results may not be optimal for the study of various disease states (i.e. Alzheimer’s disease, Parkinson’s disease). In situations where the reference region is metabolically affected by a disorder, such as in Parkinson’s disease, Alzheimer’s disease or Progressive Supranuclear Palsy, another reference region would be more appropriate. Results from the present paper are only intended for semi-quantitative FDG-PET studies involving participants who do not present with metabolic changes of pathological origin. In this spirit, we recruited relatively healthy individuals without diabetes, glucose intolerance, evidence of overt heart, liver or renal disease, cognitive impairment, smoking, or untreated dyslipidemia, hypertension, or thyroid disease. Additionally, we verified that levels of blood cholesterol were within the normal range for age. As such we strictly studied changes in brain glucose uptake that were associated with normal aging, without any comorbidities. Additionally, these participants are likely healthier on average than the normal aging population. Therefore, the results from this study may not be appropriate for individuals that have risk factors associated with cognitive decline and dementia. Further studies should examine possible references regions for various disease states. Further quantitative studies should be undertaken which include Alzheimer’s disease patients in order to validate the pons as an appropriate reference region for the study of brain glucose uptake in Alzheimer’s disease. The use of two age groups did not accurately represent the normal aging population and did not allow us to assess linearity of metabolic and volumetric changes associated with normal aging.

## Conclusions

Our goal was to identify the optimal reference region for FDG-PET studies of normal aging. To this end, we have proposed an analysis of quantitative FDG-PET using a previously validated dynamic acquisition and arterial input function technique^10^ and an image-based methodology for regions commonly used in the literature and segmented from T1-weighted MR images, including the pons, cerebellum white matter, cerebellum grey matter, cerebral white matter, cerebral grey matter, midbrain, paracentral cortex, and putamen, as well as cortical areas derived from the FreeSurfer atlas. For studies of hypometabolism using PVE and non-PVE corrected images of normal aging we recommend the use of the pons as a reference region.

## Supplementary information

Supplementary information.
