# Supplementary material for: Selection of the optimal intensity normalization region for FDG-PET studies of normal aging and Alzheimer’s disease
Source: Sci Rep. 2020 Jun 9;10:9261. doi: 10.1038/s41598-020-65957-3 (PMC7283334; doi:10.1038/s41598-020-65957-3)
Supplement: Supplementary file 1 — Supplementary information. [file 41598_2020_65957_MOESM1_ESM.docx]

**Selection of the optimal intensity normalization region for FDG-PET studies of normal aging and Alzheimer’s disease**

**Scott Nugent^1*^, Etienne Croteau^2^, Olivier Potvin^1^, Christian-Alexandre Castellano^2^, Louis Dieumegarde^1^, Stephen C. Cunnane^2^, Simon Duchesne^1,3^**

^1^ CERVO Research Centre, Quebec Mental Health Institute, Quebec, Canada

^2^ Research Center on Aging, Health and Social Sciences Center, Geriatrics Institute, Sherbrooke, Canada

^3^ Radiology and Nuclear Medicine Department, Université Laval, Québec, Canada

Supplementary Table 1

Complete list of FreeSurfer normalization regions.

| L Accumbens | L Precentral | R Lateral orbitofrontal |
| --- | --- | --- |
| L Amygdala | L Precuneus | R Lingual |
| L Caudal anterior cingulate | L Putamen | R Medial orbitofrontal |
| L Caudal middle frontal | L Rostral anterior cingulate | R Middle temporal |
| L Caudate | L Rostral middle frontal | R Pallidum |
| L Cerebellum cortex | L Superior temporal | R Paracentral |
| L Cerebellum white matter | L Superior frontal | R Parahippocampal |
| L Cuneus | L Superior parietal | R Parsopercularis |
| L Entorhinal | L Supramarginal | R Parstriangularis |
| L Fusiform | L Temporal pole | R Pericalcarine |
| L Hippocampus | L Thalamus proper | R Postcentral |
| L Inferior parietal | L Transverse temporal | R Posterior cingulate |
| L Inferior temporal | R Accumbens | R Precentral |
| L Insula | R Amygdala | R Precuneus |
| L Isthmus cingulate | R Caudal anterior cingulate | R Putamen |
| L Lateral occipital | R Caudal middle frontal | R Rostral anterior cingulate |
| L Lateral orbitofrontal | R Caudate | R Rostral middle frontal |
| L Lingual | R Cerebellum cortex | R Superior frontal |
| L Medial orbitofrontal | R Cerebellum white matter | R Superior parietal |
| L Middle temporal | R Cuneus | R Superior temporal |
| L Pallidum | R Entorhinal | R Supramarginal |
| L Paracentral | R Fusiform | R Temporal pole |
| L Parahippocampal | R Hippocampus | R Thalamus proper |
| L Parsopercularis | R Inferior parietal | R Transverse temporal |
| L Parstriangularis | R Inferior temporal | Medulla |
| L Pericalcarine | R Insula | Midbrain |
| L Postcentral | R Isthmus cingulate | Pons |
| L Posterior cingulate | R Lateral occipital | SCP |

L – left; R – right
